# T1 reduction rate with Gd-EOB-DTPA determines liver function on both 1.5 T and 3 T MRI

**DOI:** 10.1038/s41598-022-08659-2

**Published:** 2022-03-18

**Authors:** Verena Carola Obmann, Damiano Catucci, Annalisa Berzigotti, Christoph Gräni, Lukas Ebner, Johannes Thomas Heverhagen, Andreas Christe, Adrian Thomas Huber

**Affiliations:** 1 Department of Diagnostic, Interventional and Pediatric Radiology, Inselspital, Bern University Hospital, University of Bern, Freiburgstrasse 10, 3010 Bern, Switzerland; 2 Hepatology, Department of Visceral Surgery and Medicine, Inselspital, Bern University Hospital, University of Bern, Bern, Switzerland; 3 Department of Cardiology, Inselspital, Bern University Hospital, University of Bern, Bern, Switzerland

**Keywords:** Liver diseases, Liver cirrhosis

## Abstract

Magnetic resonance T1 mapping before and after Gd-EOB-DTPA administration allows quantification of the T1 reduction rate as a non-invasive surrogate marker of liver function. A major limitation of T1 relaxation time measurement is its dependency on MRI field strengths. Since T1 reduction rate is calculated as the relative shortening of T1 relaxation time before and after contrast administration, we hypothesized that the T1 reduction rate is comparable between 1.5 and 3 T. We thus compared liver T1 relaxation times between 1.5 and 3 T in a total of 243 consecutive patients (124, 1.5 T and 119, 3 T) between 09/2018 and 07/2019. T1 reduction rates were compared between patients with no cirrhosis and patients with cirrhosis Child–Pugh A-C. There was no significant difference of T1 reduction rate between 1.5 and 3 T in any patient group (p-value 0.126–0.861). On both 1.5 T and 3 T, T1 reduction rate allowed to differentiate between patients with no cirrhosis and patients with liver cirrhosis Child A-C (p < 0.001). T1 reduction rate showed a good performance to predict liver cirrhosis Child A (AUC = 0.83, p < 0.001), Child B (AUC = 0.83, p < 0.001) and Child C (AUC = 0.92, p < 0.001). In conclusion, T1 reduction rate allows to determine liver function on Gd-EOB-DTPA MRI with comparable values on 1.5 T and 3 T.

## Introduction

Assessment of liver function is important for determining the prognosis and management of patients with chronic liver disease (CLD)^1^. The degree of liver dysfunction is associated with a higher risk of developing liver failure and adverse outcome^2–4^. Liver magnetic resonance imaging (MRI) with gadolinium ethoxybenzyl-diethylenetriaminepentaacetic acid (Gd-EOB-DTPA) is frequently performed in patients with suspected liver lesions, in patients with known hepatocellular carcinoma (HCC) and in patients with unclear cholestatic, vascular or autoimmune liver disease^5^. As Gd-EOB-DTPA is a liver-specific contrast agent, it is taken up by hepatocytes through organic anion–transporting polypeptides (OATP), which are located at the sinusoidal (basolateral) membrane of human hepatocytes. After its uptake, Gd-EOB-DTPA is excreted into the bile ducts by ATP-dependent multidrug-resistant protein 2 (MRP2)^6,7^. However, before its clearance into the biliary ducts, Gd-EOB-DTPA temporarily accumulates in hepatocytes and leads to a shortening of the T1 relaxation time in the liver. T1 shortening can be visualized in the MRI as hyperintensity of the liver parenchyma in the hepatobiliary phase in contrast to possible adjacent non-hepatocyte-containing liver lesions^8^.

The degree of Gd-EOB-DTPA uptake in the liver parenchyma is strongly dependent on the number and functionality of the hepatocytes and OATP channels^9^. Several studies have demonstrated a correlation between reduced liver function and decreased hepatic Gd-EOB-DTPA uptake^10,11^ or a lower MRI relative signal intensity ratio between the liver and reference organs, such as the spleen or skeletal muscles^12,13^. Unfortunately, the use of relative signal intensity ratios is limited by a lack of precision and possible confounders such as portal hypertension influencing the splenic MRI signal or fatty degeneration of the skeletal muscles. The use of T1 mapping allows a calculation of the absolute shortening of the T1 relaxation time in the liver as a percentage without assessment of reference organs and therefore mitigates many of those limitations^14,15^.

However, in many institutions, liver MRI is performed both on 1.5 T and 3 T scanners, whereby the T1 relaxation time depends strongly on the magnetic field strength. Since the relaxivity of Gd-EOB-DTPA in plasma at 37 °C is similar at 1.5 T and 3 T (6.2 L mmol^−1^ s^−1^ at 1.5 T and 6.9 L mmol^−1^ s^−1^ at 3 T)^16^, we expected almost the same linear relationship between the T1 reduction rate and Gd-EOB-DTPA concentration in hepatocytes at 1.5 T and 3 T. Our study aimed to compare T1 relaxation times between 1.5 and 3 T in a large number of consecutive patients undergoing liver MRI.

## Methods

### Study population

**Figure 1:**
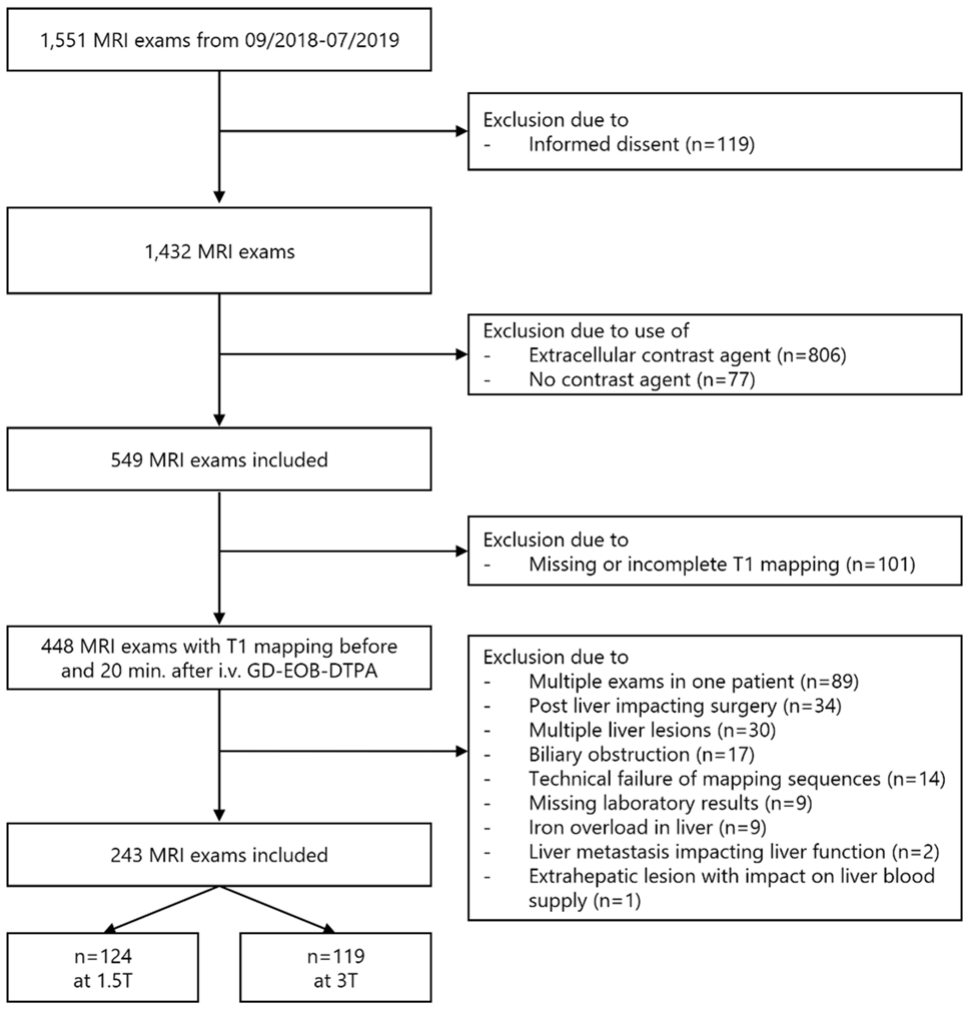
Patient flowchart. A total of 1551 consecutive liver MRI exams performed at our institution between 09/2018 and 07/2019 were included. After applying exclusion criteria, 243 exams with T1 mapping before and 20 min after Gd-EOB-DTPA administration remained: 124 acquired on a 1.5 T scanner and 119 acquired on a 3 T scanner.

In this retrospective, institutional review board-approved (Cantonal ethics committee, Bern, Switzerland, Project ID 2019-01333) and Health Insurance Portability and Accountability Act-compliant, cross-sectional comparative study, our database was screened for liver MRI exams performed in our hospital between 09/2018 and 07/2019 according to a, revealing 1,551 exams. If patients underwent two or more exams within this time period, only the first exam was included. Written informed consent to participate in research project was present from all included patients. Those patients who refused to allow their imaging data to be used for research were excluded (n = 119). Patients who underwent MRI exams that used extracellular contrast agents (n = 806) or no contrast agent (n = 77) as well as exams without T1 mapping (n = 101) were excluded. From the remaining 448 MRI exams with T1 mapping before and 20 min after intravenous Gd-EOB-DTPA administration patients were excluded due to the following reasons: repetitive exams in one patient (n = 89), liver surgery prior to MRI examination (n = 34), immeasurable liver parenchyma due to multiple liver lesions (n = 30), biliary obstruction (n = 17), technical failure of T1 mapping (n = 14), missing laboratory results (n = 9), iron overload in the liver (n = 9) and liver metastasis with impact on liver function (n = 2). Finally, 1 patient was excluded due to an extrahepatic lesion with impact on the liver blood supply. Of the resulting patient population (n = 243), 124 patients were examined on a 1.5 T scanner, and 119 were examined on a 3 T scanner (Fig. 1). For a sub-analysis, 29 additional MRI exams were analyzed in patients who underwent MRI on a 1.5 T and a 3 T scanner within 6 months. Those 29 additional MRI exams were not included in the main analysis to prevent bias and to guarantee that every patient is only represented once in the analysis.

The patients’ electronic medical records were used to determine the presence, type and extent of liver cirrhosis . Clinical information such as known diagnosis of viral hepatitis, alcohol related liver disease, non alcoholic fatty liver disease/steatohepatitis (NAFLD/NASH) or chronic biliary disease such as PSC/PBC as well as the presence of hepatic encephalopathy and ascites, and biochemical laboratory results (i.e. albumin levels, bilirubin levels, and quick values) as well as biopsy results if present within 3 months of each MRI exam were recorded and used to calculate a Child–Pugh-Score for patients with liver cirrhosis. Patients were grouped as follows: patients with no liver cirrhosis (noLC) and patients with liver cirrhosis Child A (5–6 points of the Child–Pugh score), Child B (7–9 points) and Child C (10–15 points)^17^. No liver cirrhosis was defined as no clinical history of liver cirrhosis or liver biopsy showing no liver cirrhosis.

### MRI technique

All MR exams were performed on a whole-body MRI system. A total of 124 exams were performed on a 1.5 T unit (Siemens Aera, Siemens Healthineers, Erlangen, Germany) and 119 were performed on a 3 T unit (Siemens, Skyra, Siemens Healthineers, Erlangen, Germany). Beside a routine liver MRI protocol, patients underwent T1 mapping before and 20 min after the injection of 0.25 mmol Gd-EOB-DTPA per kilogram body weight. For T1 mapping, a shortened modified Look-Locker inversion recovery (shMOLLI) single breath-hold sequence was used with an echo time of 1.01 ms, a repetition time of 740 ms, an inversion time of 225 ms and a flip angle of 35°. The field-of-view (FOV) was 306 mm × 360 mm with a matrix of 154 × 192 pixels, and 8-mm slice thickness. A total of 4 acquired slices with a breath hold each resulted in a total scan time of 1 min 37 s for the shMOLLI acquisitions. Parametric T1 maps were generated automatically on the scanner.

### Image analysis

Image analysis was performed on a clinical Picture Archiving and Communication System (PACS, IDS7, version 21.2, Sectra AB, Linköping, Sweden). Nine regions of interest (ROIs) with a minimum size of 500 pixels were drawn carefully in each liver segment (differentiating Seg IVa and Seg IVb) by three technicians from our imaging core lab, carefully corrected by a MD-PhD candidate after special training and in consensus with a board certified radiologist with 8 years of experience in liver imaging. ROI were drawn in each liver segment on the slice closest to the portal vein in the same position on the pre- and post-contrast T1 maps by avoiding large intrahepatic vessels, bile ducts and focal lesions as well as the outer liver contour to avoid partial volume effects. An average value for the liver was then calculated from the measurements of the liver segments for the native and the post-contrast T1 map (mean pre contrast T1 and mean post contrast T1). After that, The T1 reduction rate was calculated using the following formula:

$$T1 \; reduction \; rate = \frac{(mean \; pre \; contrast \; T1-mean \; post \; contrast \; T1)}{(mean \; pre \; contrast \; T1)}$$

### Statistical analysis

Statistical analysis was performed using SPSS version 25 (Armonk, New York, USA) and GraphPad Prism versions 8.0.1 and 9.0.0. (San Diego, California, USA). The T1 reduction rate was compared between the patient groups using the Kruskal–Wallis test with Dunn’s post hoc-multiple comparison test and within groups at 1.5 T and 3 T using the Mann–Whitney U test. In a pooled analysis of all patients, cutoff values of the T1 reduction rates were determined using receiver operating characteristic (ROC) analyses based on Youden’s index. Pearson’s correlation was used to compare T1 reduction rates with Child–Pugh scores in patients with cirrhosis, as well as the model of end stage liver disease (MELD) score. A power analysis for non-inferiority was performed for T1 reduction rate to differ with a maximum lower or upper boundary of 0.9 from the mean observed T1 reduction rate in every group, with a maximum standard deviation of 0.1, a power of 80% and an alpha of 0.05. Non-inferiority was assessed based on the 90%-confidence interval of the mean of the differences in every group, to determine the smallest differences between 1.5 and 3 T in every group with a p < 0.05. In a subgroup of 29 patients who underwent MRI at both 1.5 T and 3 T within 6 months, the differences between T1 reduction rates at 1.5 T and 3 T within the same patient were calculated using linear regression. Furthermore, a Bland–Altman plot was created to compare the differences between 1.5 and 3 T in these 29 patients. A p-value < 0.05 was defined as significant.

### Ethics approval

Ethical approval for this retrospective study was obtained by the local Ethics Committee of the University Hospital of Bern (Inselspital Bern).

## Results

### Patient characteristics

The 243 patients were assigned to the following groups: 62 patients with noLC, 106 with cirrhosis Child A, 56 with cirrhosis Child B and 19 with cirrhosis Child C (Table 1). Compared to patients with noLC patients with liver cirrhosis were more likely to be male, and had a higher daily alcohol consumption as well as a higher body mass index (BMI). Except for the Child C group, patients with liver cirrhosis also showed a higher prevalence of diabetes and arterial hypertension than patients with no liver cirrhosis. Further, patients with cirrhosis showed higher liver enzyme levels and lower thrombocyte levels as well as higher APRI and FIB-4 scores (p < 0.001) than noLC. The most common etiologies of liver cirrhosis were non-alcoholic- and alcohol-related liver disease with or without steatohepatitis (NAFLD/NASH, 25% and ARLD/ASH, 41%) and chronic viral hepatitis (B and C, 24%) (Table 2).

**Table 1:** Patient characteristics.

	noLC (n = 62)	Child A (n = 106)	Child B (n = 56)	Child C (n = 19)	p-value
Age, years	57 (38–72)	62 (53–70)	65 (57–71)	63 (54–69)	0.317
Male, n (%)	29 (47)	71 (67)	43 (77)	14 (74)	0.004
Alcohol consumption, n (%)	5 (8)	13 (12)	19 (34)	5 (26)	< 0.001
Diabetes, n (%)	9 (15)	33 (31)	19 (34)	3 (16)	0.037
Arterial hypertension, n (%)	18 (29)	52 (49)	29 (52)	4 (21)	0.007
BMI, kg/m^2^	23 (21–27)	27 (25–30)	28 (24–32)	26 (21–30)	< 0.001
PDFF, %	2.5 (1.7–9.6)	2.8 (1.5–9.4)	3.1 (1.9–8.4)	2.8 (2.2–4.1)	0.896
AST, U/l	25 (21–31)	40 (28–61)	52 (35–82)	59 (36–79)	< 0.001
ALT, U/l	23 (20–32)	41 (26–62)	32 (25–52)	31 (20–51)	< 0.001
GGT, U/l	26 (17–48)	92 (42–210)	152 (64–381)	95 (71–276)	< 0.001
Alkaline phosphatase, U/l	71 (59–91)	89 (69–118)	126 (89–160)	199 (134–281)	< 0.001
Bilirubin, μmol/l	7 (5–11)	11 (8–16)	17 (14–28)	43 (19–75)	< 0.001
Albumin, g/L	36 (33–38)	38 (35–40)	33 (28–35)	26 (22–27)	< 0.001
Thrombocytes, 10^9^/L	226 (189–279)	164 (118–238)	110 (71–164)	78 (57–153)	< 0.001
Quick, %	97 (85–105)	88 (80–97)	62 (57–69)	48 (38–52)	< 0.001
APRI	0.3 (0.2–0.4)	0.7 (0.4–1.2)	1.4 (0.9–2.0)	2.0 (0.5–2.8)	< 0.001
FIB-4	1.4 (0.9–1.9)	2.5 (1.5–4.3)	5.9 (3.4–8.9)	7.8 (2.5–11.2)	< 0.001
Creatinine, μmol/l	70 (61–82)	74 (65–89)	71 (57–92)	73 (57–134)	0.388

Values are presented as median and interquartile range (25%-quartile–75%-quartile) or n (%). p-values are calculated using the Kruskal–Wallis test and X^2^-test, as appropriate. Alcohol consumption was defined as ≥ 2 alcoholic beverages per day for men and ≥ 1 alcoholic beverage per day for women or the presence of a history of abusive alcohol consumption. noLC: no liver cirrhosis; Child A, B, C: Child Pugh group A, B, C; BMI: body mass index; PDFF: proton density fat fraction; AST: aspartate aminotransferase; ALT: alanine aminotransferase; GGT: gamma-glutamyltransferase; APRI: aspartate aminotransferase-to-platelet ratio index; FIB-4: Fibrosis-4 Index.

**Table 2:** Etiology liver disease cirrhosis patients.

Etiology	Child A (n = 106 )	Child B (n = 56)	Child C (n = 19)
NAFLD/NASH	30 (28%)	14 (25%)	1 (5%)
ARLD/ASH	33 (31%)	31 (55%)	11 (60%)
Viral hepatitis (B/C)	30 (28%)	12 (21%)	1 (5%)
PSC	4 (4%)	0 (0%)	1 (5%)
PBC	6 (6%)	1 (2%)	1 (5%)
Other	11 (11%)	3 (5%)	5 (26%)
Unknown	3 (3%)	2 (4%)	0 (0%)

Child A, B, C: Child Pugh group A, B, C; NAFLD: non-alcoholic fatty liver disease; NASH: non-alcoholic steatohepatitis; ARLD: Alcohol related liver disease; ASH: alcoholic fatty liver disease; PSC: primary sclerosing cholangitis; PBC: primary biliary cholangitis; Note: Multiple etiologies in one patient were possible (e.g., ASH with concurrent chronic viral hepatitis).

### Native T1 relaxation times

Native T1 relaxation times were significantly longer in liver cirrhosis groups than in noLC at 1.5 T (noLC vs. Child A with p-value of 0.007; noLC vs. Child B with p-value of < 0.001; noLC vs. Child C with p-value of < 0.001) whilst at 3 T only noLC and Child B had significantly different native T1 relaxation times (noLC vs. Child A with p-value of 0.065; noLC vs. Child B with p-value of 0.012; noLC vs. Child C with p-value of 0.104). Due to the different magnetic field strengths, native T1 relaxation times were significantly shorter at 1.5 T than at 3 T in all groups (p < 0.001) (Table 3).

**Table 3:** Native T1 relaxation times and T1 reduction rates.

	noLC	n	Child A	n	Child B	n	Child C	n	p–value
**Native T1 relaxation time, ms**									
1.5 T	583 (540–624)	26	644 (589–688)	60	688 (647–761)	27	682 (634–783)	11	< 0.001
3 T	864 (815–901)	36	935 (844–1001)	46	995 (829–1088)	29	979 (845–1222)	8	< 0.001
P-value 1.5 T vs. 3 T	< 0.001		< 0.001		< 0.001		< 0.001		
**T1 reduction rate**									
1.5 T	0.75 (0.70–0.78)	26	0.66 (0.60–0.73)	60	0.61 (0.55–0.68)	27	0.47 (0.42–0.54)	11	0.005
3 T	0.77 (0.72–0.80)	36	0.67 (0.60–0.72)	46	0.56 (0.53–0.63)	29	0.41 (0.31–0.52)	8	< 0.001
P-value 1.5 T vs. 3 T	0.126		0.861		0.267		0.600		

Values are presented as median and interquartile range (25%-quartile–75%-quartile) or n. P-values of comparisons between T1 relaxation times and T1 reduction rates of 1.5 T and 3 T were calculated using Mann–Whitney U test. P-values of group-comparisons within 1.5 T and 3 T were calculated using the Kruskal–Wallis test.

noLC: No liver cirrhosis; Child A, B, C: Child Pugh group A, B, C; IQR: Interquartile Range; ms: milliseconds.

### T1 reduction rates

**Figure 2:**
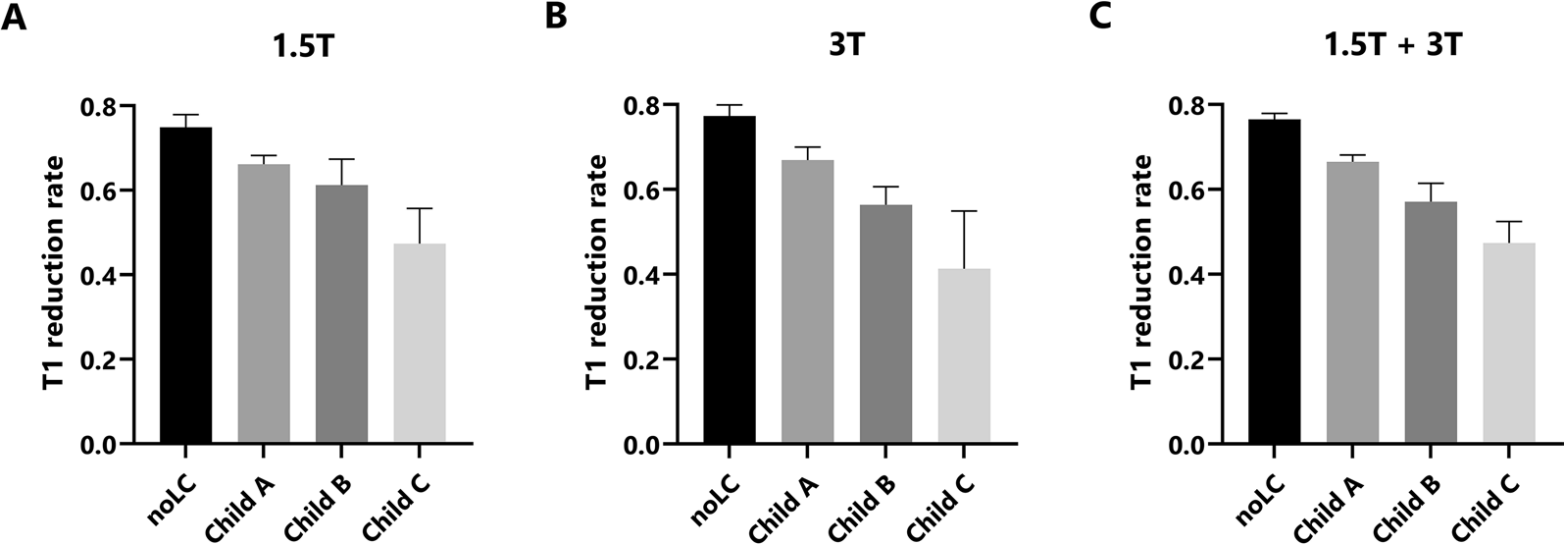
T1 reduction rates for different patient groups. The median and 95% confidence interval of T1 reduction rates on the y-axis are shown for the 4 liver groups: no liver cirrhosis (noLC) and liver cirrhosis Child–Pugh class A (Child A), class B (Child B) and class C (Child C). The results are shown for 1.5 T (**A**), 3 T (**B**) and pooled 1.5 + 3 T (**C**) data.

T1 reduction rates were significantly lower in patients with liver cirrhosis than in patients with noLC, both at 1.5 T (noLC vs. Child A with p-value of 0.033; noLC vs. Child B with p-value of < 0.001; noLC vs. Child C with p-value of < 0.001) and 3 T (noLC vs. Child A with p-value of < 0.001; noLC vs. Child B with p-value of < 0.001; noLC vs. Child C with p-value of < 0.001) (Fig. 2). There was no significant difference between the T1 reduction rates of 1.5 T and 3 T within each group (Table 3).

In a pooled analysis of the T1 reduction corrected with Dunn’s multiple comparison test, all comparisons between groups were significant with a p-value < 0.001 with the exception of the comparison between Child B and Child C, which was significant with a p-value of 0.040 (Table 4).

**Table 4:** T1 reduction rates.

	noLC (n = 62)	Child A (n = 106)	Child B (n = 56)	Child C (n = 19)
noLC (n = 62)	**0.76 (0.72–0.80)**	< 0.001	< 0.001	< 0.001
Child A (n = 106)	< 0.001	**0.67 (0.61–0.72)**	< 0.001	< 0.001
Child B (n = 56)	< 0.001	< 0.001	**0.57 (0.53–0.66)**	0.040
Child C (n = 19)	< 0.001	< 0.001	0.040	**0.47 (0.34–0.52)**

Pooled data from 1.5 to 3 T. In the diagonal (bold cells), T1 reduction rates for each liver group are presented as median and interquartile range (25%-quartile–75%-quartile). In the remaining cells, p-values calculated using Kruskal–Wallis test with Dunn’s multiple comparison corrected pairwise analysis are shown.

noLC: no liver cirrhosis; Child A, B, C: Child Pugh group A, B, C.

### Power analysis

The power analysis resulted in a minimum number of patients of n = 21 per group to show equivalence between the mean and observed values in every group with a maximum difference of 0.09, which is the minimum difference of T1 reduction rate that was observed between the groups. Based on the power analysis, the number of patients was large enough in all groups, expect the Child C group.

### Non-inferiority between the mean and observed values per group

The mean of the differences of T1 reduction rates between 1.5 and 3 T were + 0.03 for noLC (− 0.004 to + 0.059), − 0.01 for Child A (− 0.038 to + 0.016), − 0.02 (− 0.061 to + 0.029) for Child B and − 0.04 (− 0.159 to 0.070) for Child C. All difference between the mean and observed values were therefore < 0.06 for noLC, < 0.04 for Child A, < 0.07 for Child B and < 0.16 for Child C (all with p < 0.05). Except for Child C, all differences were lower than the prespecified boundary of 0.09, representing the minimum difference of T1 reduction rate between the groups.

### Correlation of the T1 reduction rate with Child–Pugh class

**Figure 3:**
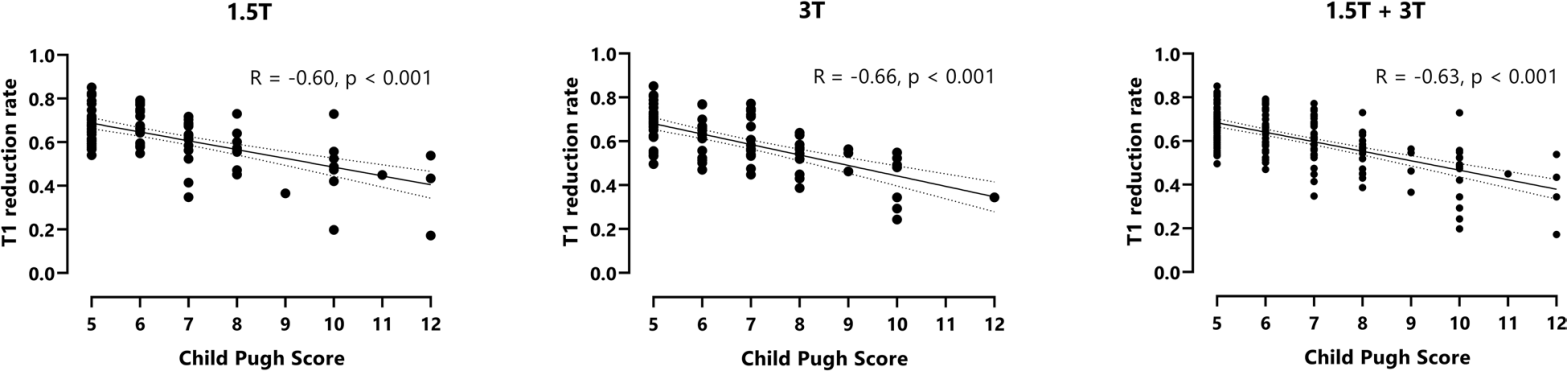
Pearson correlation between T1 reduction rate and Child–Pugh score. T1 reduction rates are shown for patients with liver cirrhosis at 1.5 T (**A**), at 3 T (**B**) and for pooled 1.5 + 3 T data (**C**). A reduced T1 reduction rate correlated moderately with a higher Child–Pugh score (R = − 0.60 at 1.5 T, R = − 0.66 at 3 T and R = − 0.63 for pooled data).

In patients with liver cirrhosis, a reduced T1 reduction rate correlated with a higher Child–Pugh score (R = − 0.60 at 1.5 T, R =  − 0.66 at 3 T and R = − 0.63 for pooled data, Fig. 3).

### Correlation of the T1 reduction rate with MELD-Score

**Figure 4:**
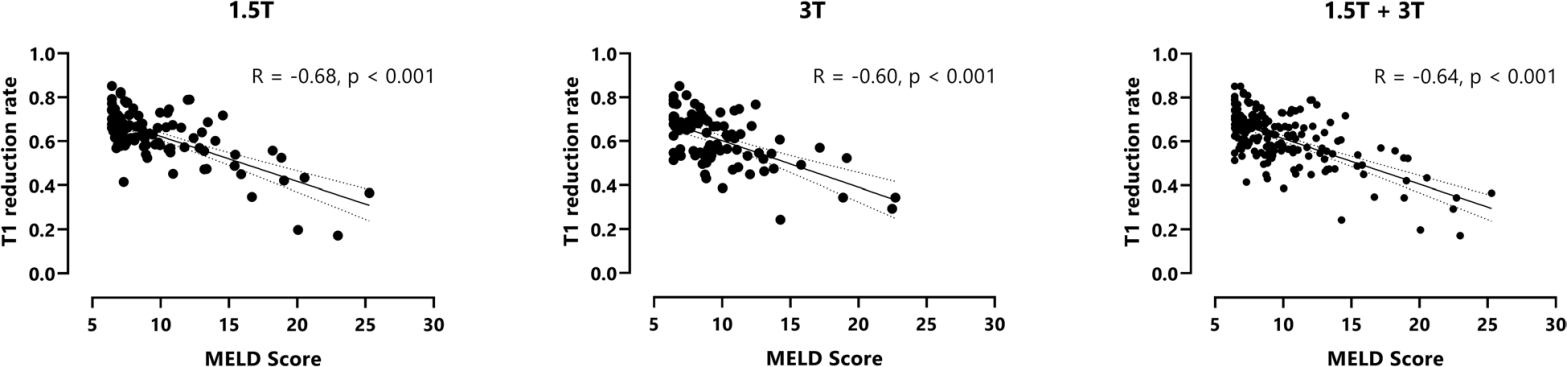
Pearson correlation between T1 reduction rate and MELD-Score. T1 reduction rates are shown for patients with liver cirrhosis at 1.5 T (**A**), at 3 T (**B**) and for pooled 1.5 + 3 T data (**C**). A reduced T1 reduction rate correlated moderately with a higher Child–Pugh score (R = − 0.68 at 1.5 T, R = − 0.60 at 3 T and R = − 0.64 for pooled data).

In patients with liver cirrhosis, a reduced T1 reduction rate correlated with a higher MELD-score (Pearson R = − 0.68 at 1.5 T, R = − 0.60 at 3 T and R = − 0.64 for pooled data, Fig. 4).

### Results of subgroup analyses

**Figure 5:**
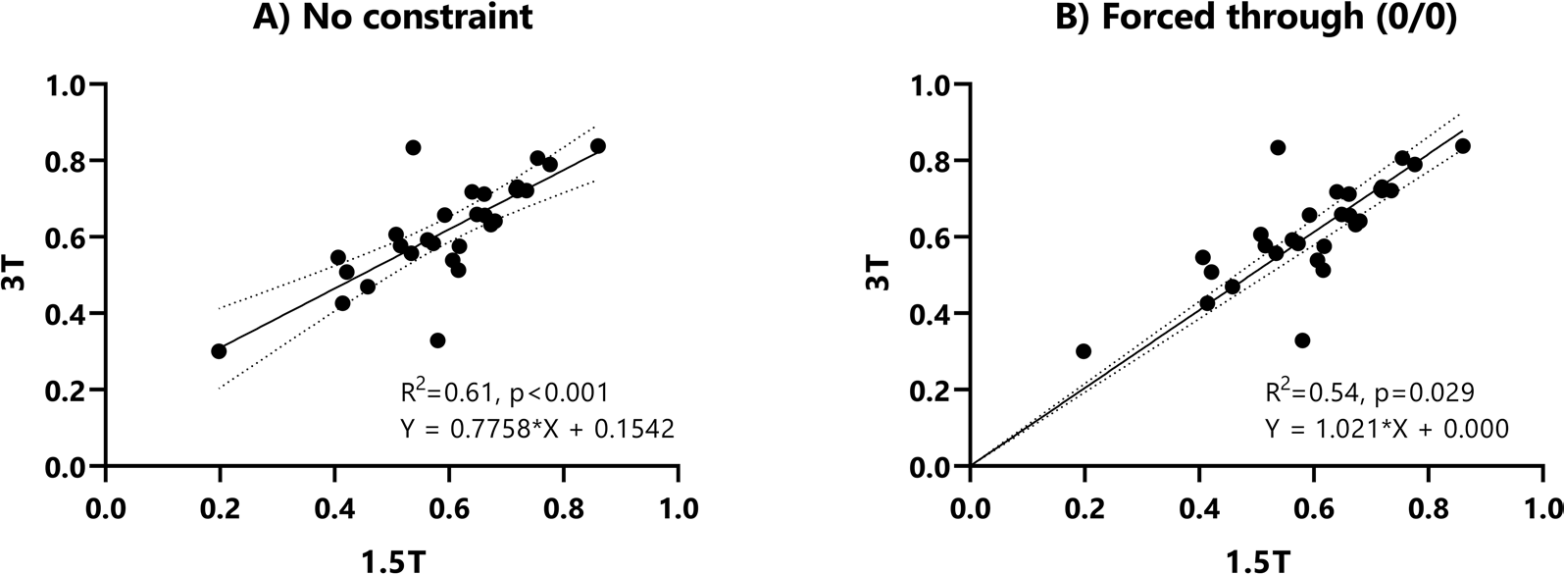
Linear regression analysis of the T1 reduction rates at 1.5 T and 3 T. This figure shows the linear regression analyses of intra-patient T1 reduction rates of 29 patients, who had MRI examinations at both 1.5 T and 3 T within 180 days apart. Without constraint, the regression formula was y = 0.78 * x + 0.15 with R^2^ = 0.61 and p < 0.001 (**A**). If the regression line was forced to go through the origin (0/0), the regression formula was y = 1.02 * x + 0.00 with R^2^ = 0.54 and p = 0.029 (**B**).

**Figure 6:**
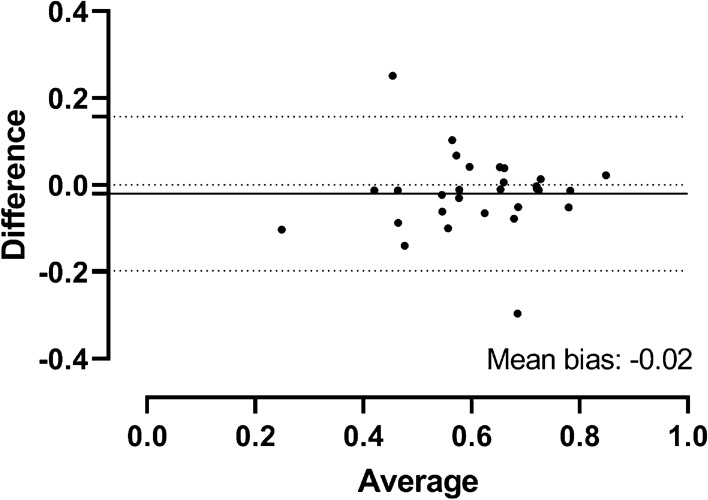
Bland–Altman analysis of the difference between 1.5 and 3 T. Bland–Altman plot for T1 reduction rates in 29 patients with both MRI at 1.5 and 3 T. The solid line shows the mean difference between T1 reduction rates at 1.5 T and 3 T. The mean bias to the zero line was − 0.02. The upper and lower dotted lines indicate the upper and lower 95% limit of agreement.

**Figure 7:**
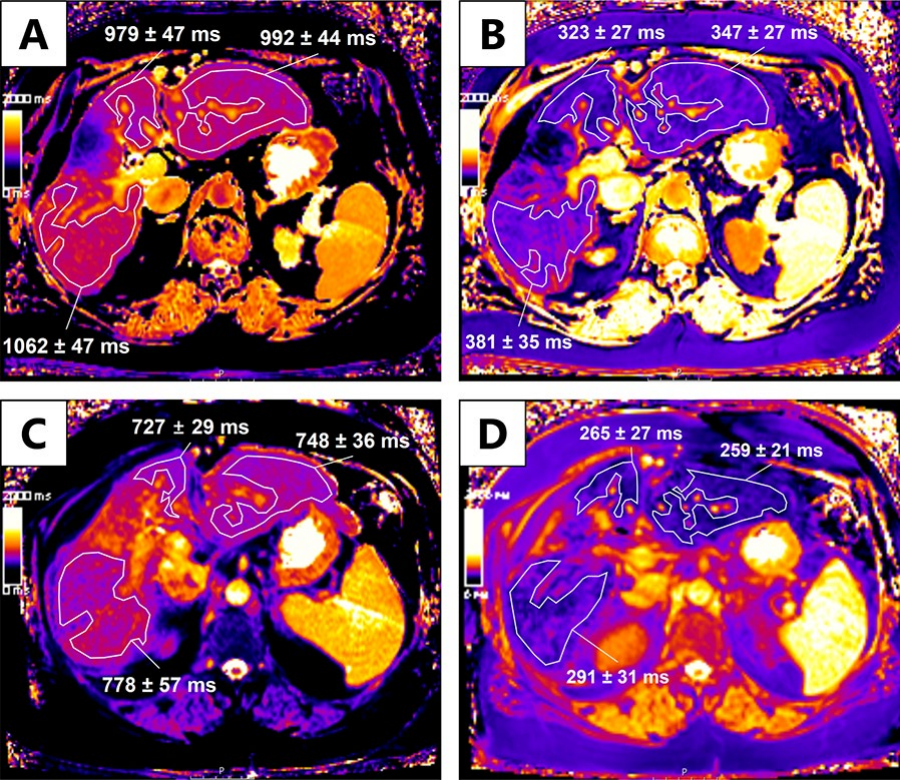
Correlation of the T1 reduction rates in a patient with exams at 1.5 T and 3 T. This figure shows images from a 54-year-old male patient with alcoholic liver cirrhosis Child A, who underwent both examination at 3 T (**A**,**B**) and 1.5 T (**C**,**D**) 160 days apart. The color bar on the left side of each figure serves as a legend for the color representation of the T1 relaxation time. Each bar ranges from 0 ms (dark) to 2000 ms (bright). In (**A**) and (**C**), measurements in the T1 map before the application of Gd-EOB-DTPA are shown. In (**B**) and (**D**), measurements in the T1 map 20 min after the application of Gd-EOB-DTPA are shown. At 3 T, we calculated a T1 reduction rate of 0.66. At 1.5 T, we calculated a T1 reduction rate of 0.65.

In a subgroup of 29 patients who underwent MRI at both 1.5 T and 3 T within a period of less than 6 months (mean 111 ± 37 days, max. 173 days), T1 reduction rates at 1.5 T and 3 T were very similar (R^2^ = 0.61, p < 0.001) (Fig. 5A). If the regression line was forced to go through the origin (0/0), the regression formula was y = 1.021 * x + 0.000 with R^2^ = 0.54 and p = 0.029 (Fig. 5B). Agreement of T1 reduction rates of 29 patients undergoing both MRI at1.5 T and 3 T are shown in the Bland–Altman Plot (Fig. 6). The mean bias between 1.5 T and 3 T was − 0.02. Comparison of the T1 reduction rates in a 54-year-old male patient with liver cirrhosis Child A, who underwent MRI examination at 1.5 T and 3 T 160 days apart, is shown in Fig. 7.

### ROC curve analysis

**Figure 8:**
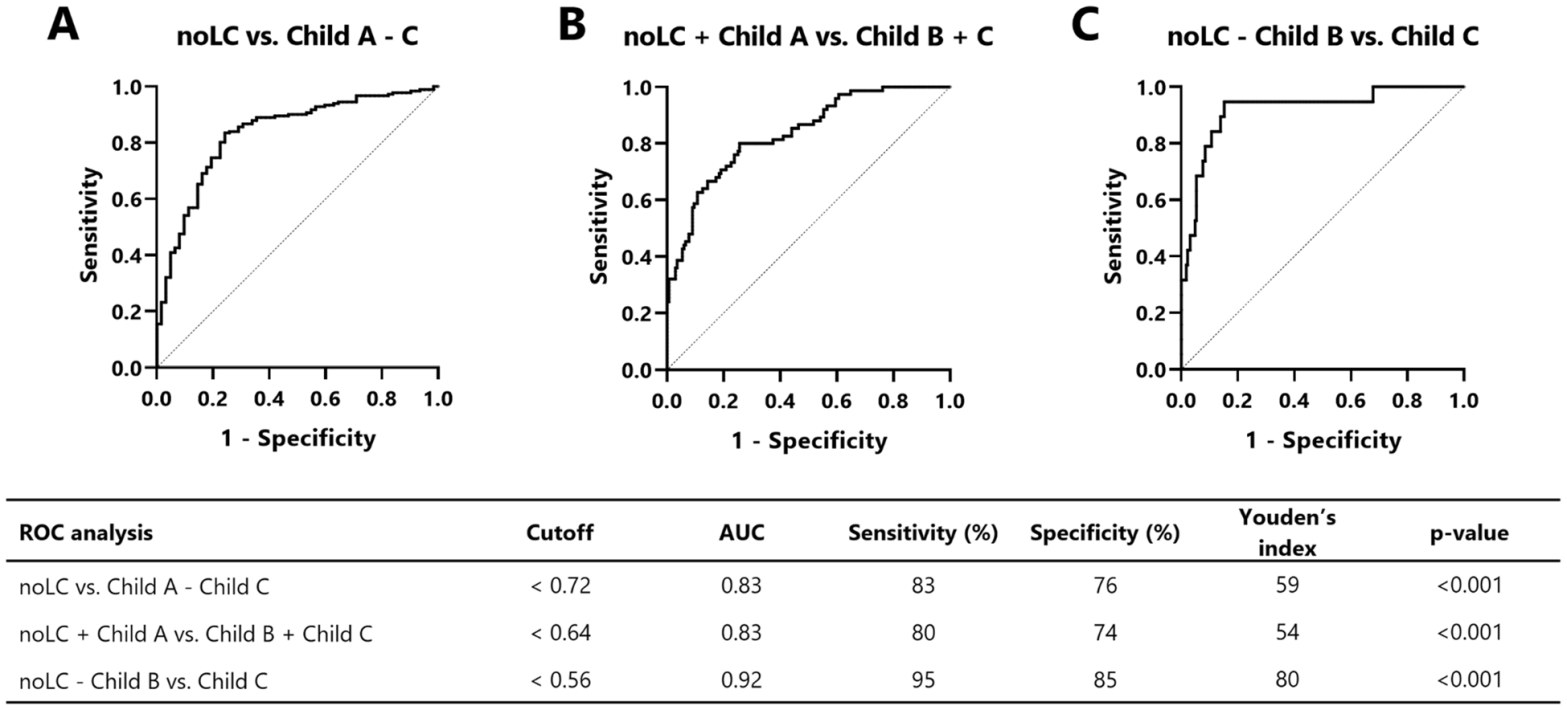
ROC analysis of pooled data. ROC curves for distinguishing between patients with noLC and liver cirrhosis on the basis of T1 reduction rates at 1.5 + 3 T are shown (**A**–**C**). AUC values for a given cutoff for each comparison as well as the sensitivity, specificity and p-values are shown. Cutoffs were determined using the Youden’s index, which is also shown. *noLC* no liver cirrhosis, *ROC* receiver operation characteristics.

AUC values, optimal cutoff values, and respective diagnostic performances for liver cirrhosis measured by the T1 reduction rate are summarized in Fig. 8. Differentiation between noLC vs. the cirrhosis groups (Child A-Child C) was possible using the T1 reduction rate with a sensitivity of 83%, a specificity of 76% and an AUC of 0.83 using a cutoff value of < 0.72. Furthermore, differentiation between noLC + Child A vs. Child B + Child C was possible with a sensitivity of 80%, a specificity of 74% and an AUC of 0.83 when using a cutoff value of < 0.64. The highest diagnostic accuracy was revealed by differentiating Child C patients from patients in all other groups with a T1 reduction rate cutoff value of < 0.56, which had a sensitivity of 95%, a specificity of 85% and an AUC of 0.92.

By using these cutoff values, the accuracy to differentiate between noLC vs. the cirrhosis groups was 81% for 1.5 T and 81% for 3 T. The accuracy for the differentiation between noLC + Child A vs. Child B + Child C was 72% for 1.5 T and 80% for 3 T. The accuracy for the differentiation between noLC + Child A + Child B vs. Child C was 90% for 1.5 T and 80% for 3 T.

## Discussion

This is the first study to show that the MRI T1 reduction rates at 1.5 T and 3 T can be used interchangeably. In contrast to the T1 relaxation time, which is dependent on the MRI field strength, the T1 reduction rate did not change between 1.5 and 3 T in different patient groups with and without liver cirrhosis. Linear regression analysis of 29 patients who underwent MRI at both 1.5 T and 3 T within 6 months revealed that the T1 reduction rates correlated significantly between the 1.5 T and 3 T scans without the need to apply any conversion factor (y = 1.02x + 0.00, p = 0.029). Bland Altman analysis showed that the T1 reduction rates were within the 95% limits of agreement except in 2 patients and the mean difference of the T1 reduction rates between 3 and 1.5 T was very low (− 0.02). The T1 reduction rate therefore represents a useful noninvasive imaging biomarker to evaluate the liver function at both 1.5 T and 3 T. The difference between the mean and observed T1 reduction rate was significantly lower than the maximum difference of T1 reduction rate between groups, except for the Child C group, where the number of included patients was too small for a statistically significant conclusion. Due to the noninvasiveness of MRI, the T1 reduction rate can be measured repetitively, allowing a longitudinal analysis of liver function, which is not possible with liver biopsy. In addition, analysis of the whole liver volume is possible, and segmental regionalities may be captured^18^.

Our results are in accordance with recently published literature (Table 5). Katsube et al. were the first to report that evaluation of hepatic uptake of Gd-EOB-DTPA using T1 mapping of the liver allows an estimation of liver function^15^. Further Lagadec et al. showed in an animal model that hepatic enhancement fraction with Gd-EOB-DTPA correlates with hepatic organic anion transporter expression^9^. However, most studies concerning this subject were conducted on 3 T scanners using the MOLLI sequence^19^, a similar look-locker sequence from another vendor (Achieva, Phillips Healthcare, Best, The Netherlands)^20,21^ and a volumetric interpolated breath-hold examination (VIBE) with variable flip-angle (FA) method^22,23^. One study by Kim et al. used both the MOLLI sequence and a B1-corrected variable FA method^24^ and obtained similar T1 reduction rate values for patients without CLD (0.71 in the study of Kim et al.) and patients with liver Cirrhosis Child B or C (0.45 in the study by Kim et al.) as we did. Another study by Yoon et al., who used the MOLLI sequence for T1 mapping, showed a similar T1 reduction rate as the one we found in liver cirrhosis Child A patients (Yoon et al. Child A = 0.63), while the T1 reduction rates of patients with advanced cirrhosis were slightly lower than those of our study population (Yoon et al. Child B7 = 0.51, Child B8 = 0.45 and Child C10 = 0.34). This might be explained by the small number of samples in their advanced cirrhosis groups^19^. The results obtained with look-locker techniques from other vendors (Achieva, Phillips Healthcare, Best, The Netherlands) are comparable with our results, showing a T1 reduction rate of 0.71 in healthy volunteers (Liu et al.)^20^. Furthermore, studies that used a different variable flip-angle method showed a T1 reduction rate of 0.67 in patients without CLD^24^, which is therefore comparable with our result. Only one study, which was performed by Yang et al., used T1 mapping at 1.5 T using a dual flip-angle VIBE sequence (Magnetom Aera, Siemens Healthineers, Erlangen, Germany), and obtained a similar T1 reduction rate of 0.70^25^. Similarly, other studies analyzed the relative enhancement, as well as the liver to spleen and the liver to muscle contrast ratio and similarly found no significant differences between 1.5 and 3 T^26,27^.

**Table 5:** T1 reduction rates comparison.

	Technique	Field strength	Patient group	T1 reduction rate
Yoon et al.	MOLLI	3 T	Child A	0.63
			Child B8	0.51
			Child B9	0.45
			Child B10	0.34
Liu et al.	Look locker other vendor	3 T	Volunteers	0.71
			Child A	0.71
			Child B-C	0.54
Pan et al.	Look locker other vendor	3 T	F0	0.94
			F1	0.89
			F2	0.94
			F3	0.71
			F4	0.52
Haimerl et al.	VIBE variable FA	3 T	F0	0.69
			F1	0.51
			F2	0.55
			F3	0.42
			F4	0.39
Kim et al.	MOLLI	3 T	Normal liver	0.71
			CLD	0.69
			Child A	0.62
			Child B-C	0.45
	B1 corrected variable FA	3 T	Normal liver	0.67
			CLD	0.64
			Child A	0.58
			Child B-C	0.44
Yang et al.	VIBE variable FA	1.5 T	F0	0.71
			F1	0.71
			F2	0.66
			F3	0.65
			F4	0.65

MOLLI: modified look locker inversion recovery sequence, FA: flip angle, VIBE: volumetric interpolated breath-hold examination, CLD: chronic liver disease, Child A, B, C: Child Pugh group A, B, C.

The native T1 relaxation time of the liver was longer at 3 T, compared with 1.5 T, while patients with liver cirrhosis showed longer native T1 relaxation times than patients without liver cirrhosis, which is consistent with earlier publication^28^. One possible explanation for the longer T1 relaxation time in cirrhosis is the deposition of collagen fibers in the extracellular space, resulting in longer T1 relaxation time, as it is also known from other organs such as the myocardium or skeletal muscles^29^. Another possible explanation is that the T1 relaxation time could be prolonged not only because of fibrosis, but also because of inflammation edema in patients with CLD^28^. In our study, the T1 reduction rate was able to discriminate between different patient groups much better than native T1. Whether the combination of native T1 and T1 reduction rate yields an incremental value to differentiate between patients with different degrees of fibrosis, inflammation and loss of liver function warrants further investigation.

The noninvasive gold standard to grade liver fibrosis is MR elastography, which measures liver stiffness based on acoustic shear waves generated by an external driver^30^. ROC analysis revealed that the predictive value of the T1 reduction rate to discriminate between patients with noLC and patients with liver cirrhosis (AUC = 0.83) was slightly lower in our study than in MR elastography studies^31^. For example, Singh et al. received an AUC for such a comparison of F0 vs. F1–F4 = 0.86 and F0–F3 vs. F4 = 0.91^31^. Nevertheless, the T1 reduction rate may represent a useful and easily available MRI-based method to grade liver function when MR elastography equipment is not available. Along with MR elastography, which assesses liver stiffness and not hepatocellular function, liver T1 function mapping may be valuable for combined noninvasive MRI-based assessment of liver fibrosis and liver function mapping.

## Limitations

We acknowledge that our study has limitations, mainly related to its retrospective nature. Due to the cross-sectional design, a relatively small sample size of Child C patients (n = 19) was used, while the number of patients in all other groups was much larger. The inclusion of a broad spectrum of chronic liver disease etiologies in a cross-sectional study might have led to confounding of the measured T1 relaxation times, especially in the presence of fat but realistically represents the setting of a university hospital radiology department. Further, the interest of the study was not to measure and compare absolute T1 relaxation times of the patients but the relative change of T1 relaxation over time after Gd-EOB-DTPA administration.

## Conclusion

This study shows that the T1 reduction rate allows to determine liver function on Gd-EOB-DTPA MRI with comparable values on 1.5 T and 3 T.

## Data Availability

Data generated for analysis during this study are included in this published article. Original patient data files are precluded from dissemination following Swiss Federal Law regulations (https://www.admin.ch/opc/de/officialcompilation/ 2013/3381.pdf). Data requests may be sent to: Kantonale Ethikkommission für die Forschung Murtenstrasse 31, 3010 Bern (Tel. + 41 31 633 70 70, Fax + 41 31 633 70 71, info.kek.kapa@gef.be.ch).

## Abbreviations

ALT: Alanine aminotransferase
ARLD/ASH: Alcohol related liver disease/alcoholic steatohepatitis
AST: Aspartate-aminotransferase
AUC: Area under the curve
BMI: Body mass index
CLD: Chronic liver disease
Gd-EOB-DTPA: Gadolinium ethoxybenzyl-diethylenetriaminepentaacetic acid
GGT: Gamma-glutamyl-transpeptidase
HCC: Hepatocellular carcinoma
MELD: Model of end stage liver disease
MOLLI: Modified Look-Locker inversion recovery sequence
MRI: Magnetic resonance imaging
NALFD/NASH: Non-alcoholic fatty liver disease/nonalcoholic steatohepatitis
noLC: No liver cirrhosis
OATP: Organic anion-transporting polypeptides
PDFF: Proton density fat fraction
ROC: Receiver operating characteristics
ROI: Region of interest
shMOLLI: Shortened modified Look-Locker inversion recovery sequence
VIBE: Volumetric interpolated breath-hold examination
